# Nonspecific Effects of Infant Vaccines Make Children More Resistant to *SARS-CoV-2* Infection

**DOI:** 10.3390/children9121858

**Published:** 2022-11-29

**Authors:** Luis Fonte, María Ginori, Gissel García, Yisel Hernández, Yaxsier de Armas, Enrique J. Calderón

**Affiliations:** 1 Department of Parasitology, Institute of Tropical Medicine “Pedro Kourí”, Havana 11400, Cuba; 2 Department of Teaching, Polyclinic “Plaza de la Revolución”, Havana 11300, Cuba; 3 Department of Pathology, Hospital “Hermanos Ameijeiras”, Havana 10300, Cuba; 4 Department of Vector Control, Institute of Tropical Medicine “Pedro Kourí”, Havana 11400, Cuba; 5 Department of Clinical Microbiology Diagnostic, Hospital Center of Institute of Tropical Medicine “Pedro Kourí”, Havana 11400, Cuba; 6 Department of Pathology, Hospital Center of Institute of Tropical Medicine “Pedro Kourí”, Havana 11400, Cuba; 7 Instituto de Biomedicina de Sevilla, Hospital Universitario Virgen del Rocío/Consejo Superior de Investigaciones Científicas/Universidad de Sevilla, 41013 Seville, Spain; 8 Centro de Investigación Biomédica en Red de Epidemiología y Salud Pública (CIBERESP), 28029 Madrid, Spain; 9 Depatamento de Medicina, Facultad de Medicina, Universidad de Sevilla, 41009 Sevilla, Spain

**Keywords:** COVID-19, *SARS-CoV-2*, children susceptibility, children immune competence, children vaccines

## Abstract

A myriad of reasons, or a combination of them, have been alluded to in order to explain the lower susceptibility of children to *SARS-CoV-2* infection and the development of severe forms of COVID-19. This document explores an additional factor, still little addressed in the medical literature related to the matter: nonspecific resistance to *SARS-CoV-2* that could be generated by vaccines administered during childhood. The analysis carried out allows one to conclude that a group of vaccines administered during childhood is associated with a lower incidence and severity of *SARS-CoV-2* infection among pediatric ages. Looking from an epidemiological perspective, this conclusion must be taken into consideration in order to ensure greater rationality in the design and implementation of prevention and control actions, including the administration of the COVID-19 vaccine, for these ages.

## 1. Introduction

*Severe Acute Respiratory Syndrome Coronavirus 2 (SARS-CoV-2)* infection activates innate and adaptive immune responses that, in the most common and benign of evolutions, lead to the containment of the viral replication at the gateway to the host (the highest portions of the respiratory system), and, in the least frequent and most unfavorable of the sequences, after allowing the virus to descend to the lower portions of the respiratory system, stimulate an intense pulmonary inflammatory reaction that, leading to more severe complications, can end in death [1].

In general, children pass through the most favorable end of the clinical spectrum mentioned in the previous paragraph. Unlike other infectious diseases, such as malaria and the common flu, in which children exhibit higher incidence and mortality rates than older people, *SARS-CoV-2* infection among pediatric ages is less frequent and relatively benign, as it occurs with many childhood diseases, for example, herpesviruses (VZV and EBV), which tend to be worse when the first infection is in adulthood [2,3,4,5,6,7,8,9]. Similar behaviors were recorded on the occasion of infections caused by *SARS-CoV* and *Middle East Respiratory Syndrome Coronavirus (MERS-CoV)*, the coronaviruses that caused two pandemics at the beginning of this century [10,11]. Furthermore, children also appear to be less likely to transmit *SARS-CoV-2* infection than their adult counterparts [12].

A myriad of reasons, or a combination of them, have been put forward to explain the lower susceptibility of minors to *SARS-CoV-2* infection and to the development of severe forms of coronavirus disease of 2019 (COVID-19). These explanations have been classified into two groups: protective factors present in pediatric ages and risk factors that increase with age [5].

Among the protective factors present in pediatric ages, the following are mentioned as explanations: (i) Lower intensity of exposure to *SARS-CoV-2* (since the family dynamics established during the pandemic are intended to protect the youngest) [13]; (ii) Higher steady-state expression of interferon (IFN) response genes in the airway epithelium of children, which can result in less viral spreading (however, if this factor was broadly significant, children should be more resistant to many respiratory viruses, but this is not the case because, for example, children are more sensitive to influenza A virus) [14]; (iii) Better functioning immune mechanisms, especially those of innate immunity (for example, better containment of infectious processes by natural antibodies) [3]; (iv) Higher frequency of recurrent and concurrent viral infections (these infections can induce a state of activation of the innate immune system, which includes the epigenetic changes that characterize the training of innate immunity) [15,16]; (v) Modulation of the inflammatory component of host immune responses to helminthic infections, which is more frequent among pediatric ages [17]; (vi) Gut microbiota changes with age, which potentially provide a better defense against infection (for instance, children generally have higher numbers of Bifidobacterium than adults) [18]. Children may also have a higher nasopharyngeal colonization of viruses and bacteria, which by competition may limit the growth of *SARS-CoV-2* and lead to a reduced colonization of the pathogen [8].

On the other hand, while children have fewer exposures to common coronaviruses, those do not provide long-term immunity; thus, adults do not have a large immunological advantage of re-exposure [19].

Among the risk factors that increase with age, the following are described as explanations: (i) Alterations in endothelial functions and coagulation present in older people [20]; (ii) Changes in the density and affinity of the enzyme converting angiotensin 2 (ACE2) and the transmembrane serine protease 2 enzyme in epithelial cells of the mucosa of the respiratory system [21]; (iii) The systemic immune response in blood is characterized by a naiver state in children (on the contrary, adults show a highly cytotoxic immune compartment in the blood, probably due to the failure to restrict viral spread, and this elevated systemic response in adults can lead to widespread damage to immune organs) [22]; (iv) The presence of T cells and anti-coronavirus antibodies, a consequence of previous infections, which could be related to adverse antibody-mediated amplification phenomena [23]; (v) Increased immunosenescence and chronic inflammation [24]; (vi) Higher prevalence of comorbidities [25]; (vii) Acquisition of memory T and B cells during childhood and adulthood, combined with reduced thymic output, shifts the adaptive immune system into a compartment based on memory in aged individuals (this reduces the pool of unique immune receptors within naive lymphocytes, making it less likely that a high affinity immune receptor is directly available against *SARS-CoV-2* antigens) [26,27]; (viii) Levels of melatonin, which is an indoleamine hormone produced mainly by the pineal gland, are negatively correlated with age [28,29]. Infection with *SARS-CoV-2* can result in a high production of neutrophil myeloperoxidase (MOP) and reactive oxygen species (ROS), both of which are involved in the combat of pathogens, but can cause tissue damage if this response is too strong. Melatonin can inhibit MOP activity, as well as ROS scavengers, potentially reducing the severity of COVID-19 [30].

Here, we refer to an additional factor, still little addressed in the medical literature related to the subject: the nonspecific resistance to *SARS-CoV-2* that could be generated by vaccines administered during childhood.

## 2. Nonspecific Effects of Vaccines

Old and new evidence shows that vaccines against infections have nonspecific consequences on the ability of the recipient’s immune system to control other pathogens (these are now referred to as nonspecific or heterologous effects) in addition to the specific effects on the entities for which they were designed. Here are the first three pieces of evidence mentioned in chronological order: (i) At the beginning of the nineteenth century, immunization against smallpox, the first vaccine used in humans, made it clear that it protected not only against that virosis but also against conditions as diverse as atopic diseases, measles, scarlet fever, and syphilis [31]; (ii) When the vaccine against tuberculosis, also known as BCG (Bacillus Calmette-Guerin) was introduced in Sweden more than 80 years ago, subsequent mortality was almost three times lower in immunized children, a reduction much greater than expected based solely on the reduction in the number of tuberculosis deaths in immunized infants [5]; (iii) During the last two decades, several studies have shown that in areas where children have been immunized against measles, infant mortality figures are lower than expected due to the prophylactic effects of the vaccine on the virus [32].

Recent research suggests that the nonspecific effects of vaccines are produced basically owing to two mechanisms [33]. *Grosso modo*, these works in the following ways:

(i) In response to antigens contained in the vaccine preparation, CD4 and CD8 memory cells with cross-reactivity with antigens from other pathogens are generated, and, in the event of new contact with one of these other pathogens, a rapid activation of cross-reactivity memory cells occurs. Those cells, via mobilizing effector humoral and/or cellular components, act on the infectious process generated by the nonvaccine pathogen [33].

(ii) In response to components of the vaccine preparation, innate immune cells (monocytes, macrophages, and natural killer cells) are sensitized. Several types of pattern recognition receptors (PRRs) on the surface of these cells recognize pathogen-associated molecular patterns (PAMPs). It has been suggested that an increase in the expression of PRRs is responsible, at least in part, for the sensitization of innate immunity. In addition, recent studies show that innate immune responses, especially after repeated stimuli, such as those produced after the use of live vaccines, also exhibit adaptive characteristics that can contribute to protection against subsequent infections caused by pathogens other than those targeted by the vaccine, a process that occurs fundamentally through metabolic changes and epigenetic reprogramming mechanisms and that has been termed the “training of innate immunity” [33].

A growing body of evidence suggests that some vaccines administered during childhood could confer resistance to *SARS-CoV-2* infection through at least one of these immune mechanisms and, consequently, promote a more attenuated clinical expression of the virus among pediatric ages [5]. In the following, we briefly refer to those areas for which the most solid evidence has been accumulated.

### 2.1. Bacillus Calmette-Guerin (BCG) Vaccine

Many countries promote the vaccination of newborns with BCG, a live attenuated strain of *Mycobacterium bovis* that is effective in preventing tuberculosis and leprosy [34]. Most of these countries have been reported to show lower rates of *SARS-CoV-2* infection incidence and COVID-19 mortality than countries such as Italy, Spain, and the United States, where BCG is rarely administered [35]. Ongoing controlled studies, some of which are conducted by the World Health Organization, seek to support the epidemiological evidence mentioned above.

In the sense that the information available today allows us to conclude, there are two mechanisms by which immunization with BCG would reduce the incidence of *SARS-CoV-2* infection and the severity of COVID-19: (i) BCG, like other live vaccines, induces metabolic and epigenetic changes that enhance innate immune responses to subsequent infections, a typical example of training innate immunity [36], and (ii) a protein from *M. bovis*, which has a significant degree of homology with another protein in the capsid of *SARS-CoV-2*, induces the production of cross-reactive antibodies against this virus protein, which is essential for its infectivity [37].

### 2.2. Diphtheria-Tetanus-Pertussis (DTP) Vaccine

Diphtheria (D) and tetanus (T) vaccines contain inactivated toxins (toxoids) produced by *Corynebacterium diphtheriae* and *Clostridium tetani*, respectively. In the combined diphtheria-tetanus-pertussis (DTP) vaccine, the D and T components are combined with *Bordetella pertussis* antigens (P antigen). DTP is available in two main formulations: DTaP and DTwP. The first contains selected cell-free *B. pertussis* antigens (aP, acellular pertussis) and the second includes inactivated whole *B. pertussis* cells (wP, whole pertussis) [38].

Recently, a study on the amino acid sequences of some microorganisms and vaccines found that DTP, particularly its D and T components, has a significant cross-reaction potential with *SARS-CoV-2*. The cross-reactive epitopes of DTP with *SARS-CoV-2* correspond to both B lymphocytes and CD4 and CD8 T cells, including cytotoxic T cells. In most countries, children are immunized with DTP three times during the first year of life and at the age of 6 years. Consequently, it has been speculated that children could also be protected against *SARS-CoV-2* infection by DTP-induced cross-reactive immunity [39].

Another fact called for attention in the mentioned study: In the DTaP vaccine, P antigens do not contribute toward increasing the cross-reactive immunity provided by the D and T components; however, in DTwP, wP provides as much cross-reactive immunity as the D and T antigens combined. This observation suggests that the DTwP vaccine may confer more protection against *SARS-CoV-2* than DTaP. In Europe and Asia, following the current trend, most countries use DTaP. Interestingly, the incidence and severity of *SARS-CoV-2* infection are lower in the European and Asian countries that made up the former Soviet Union, where DTwP is still used [39].

### 2.3. Measles Vaccine

As mentioned above, in areas where children have been immunized against measles, global infant mortality figures are lower than expected due to the prophylactic effects of the vaccine on the virosis [32]. A study conducted in 2008 showed that immunization with a measles vaccine induced the production of effectors of acquired immunity, both humoral and cellular, against *SARS-CoV* [32]. An epidemiological survey conducted during the first wave of the ongoing pandemic found that the Chinese child population, with a higher level of immunization against measles than the Italian counterpart population, was infected with *SARS-CoV-2* with less frequency and severity [40]. The authors of that work considered that the difference was due to structural similarities between the measles virus and *SARS-CoV-2*, leading to cross-reactions between the two [40]. Furthermore, other authors believe that the possible protective effects of measles immunization against *SARS-CoV-2* infection could be due, as in the cases of oral immunization against poliomyelitis and other live vaccines, to training the host’s innate immunity [5].

### 2.4. Rubella Vaccine

Franklin et al. demonstrated that macrodomains of *SARS-CoV-2*, the rubella virus, and its component in the PRS vaccine (Mumps, Rubella, and Measles) vaccine have a 29% amino acid identity [8]. This finding suggests that both viruses and the corresponding component in PRS share at least one protein fold. This could be the reason why people who reach the highest levels of *SARS-CoV-2,* and consequently are more likely to develop severe COVID-19, produce higher levels of antirubella IgG (161.9 + 147.6 IU /mL) compared to those with lower infections and milder disease (74.5 + 57.7 IU/mL). This being the case, it could also happen the other way around; that is, antibodies against *SARS-CoV-2* could also circulate in individuals immunized against the rubella virus [4].

In addition to the mechanism of acquired immunity described in the previous paragraph, other authors believe that the possible protective effects of rubella immunization against *SARS-CoV-2* infection, like other live vaccines, could also be due to the training of innate host immunity [41].

### 2.5. Hepatitis a Vaccine

In Africa, Asia, and parts of Central and South America, where the incidence of hepatitis A virus infection is high and vaccination against it is very common, the seroprevalence of antibodies to the virus is close to 100%. Consequently, in Europe and the United States, where the incidence of hepatitis A virus infection is lower and vaccination against it is rare, the seroprevalence of antibodies against the virus is very low [42]. Since the first wave of the COVID-19 pandemic, it has become evident that *SARS-CoV-2* infection shows a lower incidence and severity in geographic areas where the seroprevalence of antibodies against hepatitis A virus is higher, such as in Africa, Asia, and areas of Central and South America [43]. Sarialioglu et al., analyzing these data, consider that both natural infection with hepatitis A virus and vaccination against it protect against *SARS-CoV-2* infection and the development of severe symptoms of COVID-19 through an antibody-mediated cross-reaction mechanism. The aforementioned authors hypothesize that individuals who have suffered natural infection with hepatitis A virus or have been immunized against it develop mucosal antibodies against it that, cross-reacting with *SARS-CoV-2*, limit mucous colonization and thus reduce its descent into the lower respiratory tract and the development of more serious clinical complications [43].

### 2.6. Oral Polio Vaccine (OPV)

Various studies have shown the effectiveness of oral polio vaccine (OPV) against respiratory infections [44] and enteric infections [45,46]. Considering that *SARS-CoV-2* predominantly infects cells in the respiratory and intestinal tract, where ACE2 receptors are present, a heterologous protective effect of OPV against COVID-19 has been suggested [35].

## 3. Conclusions

When almost three years have passed since the infection of humans with a new coronavirus was documented in the Chinese city of Wuhan, one fact continues to attract the attention of the scientific community faced with the diagnosis, treatment, and control of the new virus: children have a lower susceptibility to *SARS-CoV-2* infection and to the development of severe forms of COVID-19. In this work, we have delved into an aspect that, possibly in combination with other factors mentioned in the introduction of this document, could be contributing to resistance to *SARS-CoV-2* infection and to the attenuation of its clinical expression among early ages. The analysis carried out allows one to conclude that a group of vaccines administered during childhood is associated with a lower incidence and severity of *SARS-CoV-2* infection among pediatric ages. Looking from an epidemiological perspective, this conclusion must be taken into consideration in order to ensure greater rationality in the design and implementation of prevention and control actions, including the administration of the COVID-19 vaccine, for these ages.

## Data Availability

Not applicable.
